# Management of Penetrating Skull Base Injury: A Single Institutional Experience and Review of the Literature

**DOI:** 10.1155/2017/2838167

**Published:** 2017-07-30

**Authors:** Danfeng Zhang, Jigang Chen, Kaiwei Han, Mingkun Yu, Lijun Hou

**Affiliations:** Department of Neurosurgery, Shanghai Institute of Neurosurgery, Shanghai Changzheng Hospital, 415 Fengyang Road, Shanghai 200003, China

## Abstract

**Background:**

Penetrating skull base injury (PSBI) is uncommon among head injuries, presenting unique diagnostic and therapeutic challenges. Although many cases of PSBIs have been reported, comprehensive understanding of its initial diagnosis, management, and outcome is still unavailable.

**Materials and Methods:**

A retrospective review was performed for patients treated in neurosurgical department of Changzheng Hospital for PSBIs. Presurgical three-dimensional (3D) Slicer-assisted reconstructions were conducted for each patient. Then we reviewed previous literature about all the published cases of PSBIs worldwide and discussed their common features.

**Results:**

A total of 5 patients suffering PSBIs were identified. Penetrating points as well as the surrounding neurovascular structures were clearly visualized, assisting in the presurgical planning of optimal surgical approach and avoiding unexpected vascular injury. Four patients underwent craniotomy with foreign bodies removed successfully and 1 patient received conservative treatment. All of them presented good outcomes after proper management.

**Conclusion:**

Careful physical examination and radiological evaluation are essential before operation, and angiography is recommended for those with suspected vascular injuries. 3D modeling with 3D Slicer is practicable and reliable, facilitating the diagnosis and presurgical planning. Treatment decision should be made upon the comprehensive evaluation of patient's clinicoradiological features and characteristics of foreign bodies.

## 1. Introduction

Penetrating injuries of the skull base caused by foreign bodies are relatively uncommon, representing about 0.4% of head injuries [1, 2]. Various foreign bodies have been reported in penetrating skull base injuries (PSBIs), including wood, bamboo, metallic fragments, and toothbrushes. PSBIs could present unique diagnostic and therapeutic challenges. Patients may be initially asymptomatic but subject to serious events for several days, months, or even years after the injuries [3]. It is not difficult to detect most of intracranial foreign bodies by head computed tomography (CT) scan. However, the relationships between foreign bodies and its surrounding structures are hard to decide, which is the prerequisite for the management of these injuries [4, 5].

Treatment for PSBIs includes the surgical retraction of foreign bodies, prevention of infection, management of vascular injuries, reconstruction of skull base, and so forth [3, 5–7]. Due to the low incidence, there is no sufficient literature about the diagnosis and management of these patients. The aim of our study is to present patients of PSBIs treated in our department and review related literature in order to highlight the proper management of PSBIs and improve prognosis in the long run.

## 2. Materials and Methods

A retrospective review was conducted for patients who were treated in our hospital between January 2010 and September 2016 for PSBIs. This study was performed in accordance with the Declaration of Helsinki (1964) and approved by the investigational review board of Changzheng Hospital. Informed consent was available for each patient. Presurgical head CT scans were performed for all patients. Moreover, digital subtraction angiography (DSA) was conducted on suspicion of vascular injuries and magnetic resonance imaging (MRI) was performed in case of nonmetallic objects. Data concerning patients' demographics, mechanisms of injury, medical managements, complications, and prognosis were collected by two authors (D. F. Zhang and J. G. Chen).

**Figure 1:**
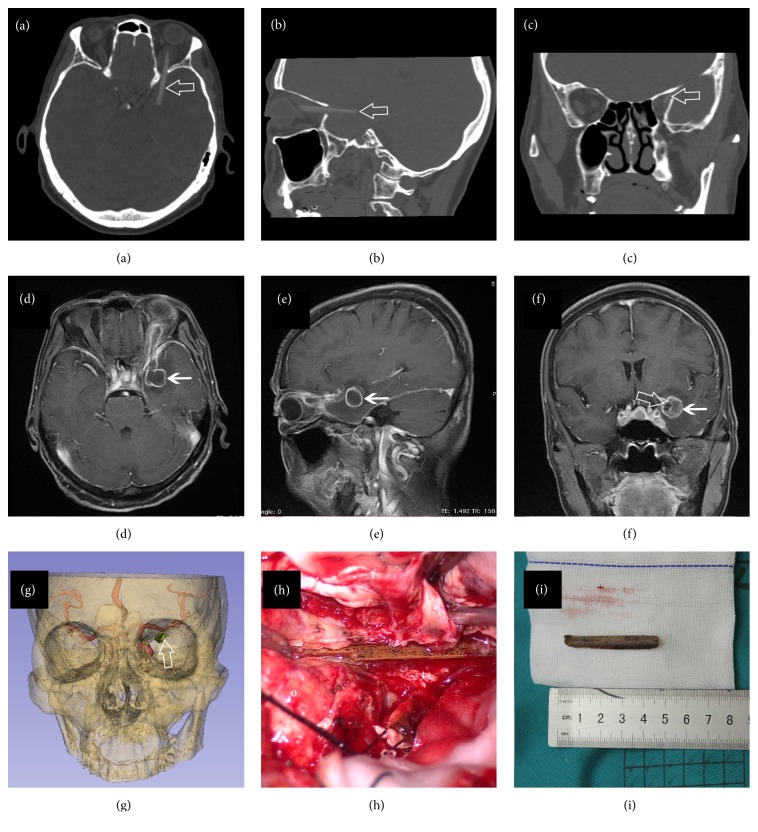
Head CT scan demonstrated a bamboo stick (hollow arrow, ⇦) penetrating into the temporal lobe via superior orbital fissure. The bamboo stick presented as high density on the CT scan (a, b, c). Contrast enhanced MRI revealed an abscess (simple arrow, *←*) around the bamboo stick in temporal lobe (d, e, f). 3D reconstruction of the skull, cerebral artery, and bamboo stick (hollow arrow, ⇦) was performed by 3D Slicer software to visualize the relationship among these structures (g). Intraoperative photography (h) displayed the bamboo stick in original place (o, orbital side; f, frontal side; t, temporal side). Photography showed the removed bamboo stick (i). 3D, three-dimensional; CT, computed tomography; MRI, magnetic resonance imaging.

**Figure 2:**
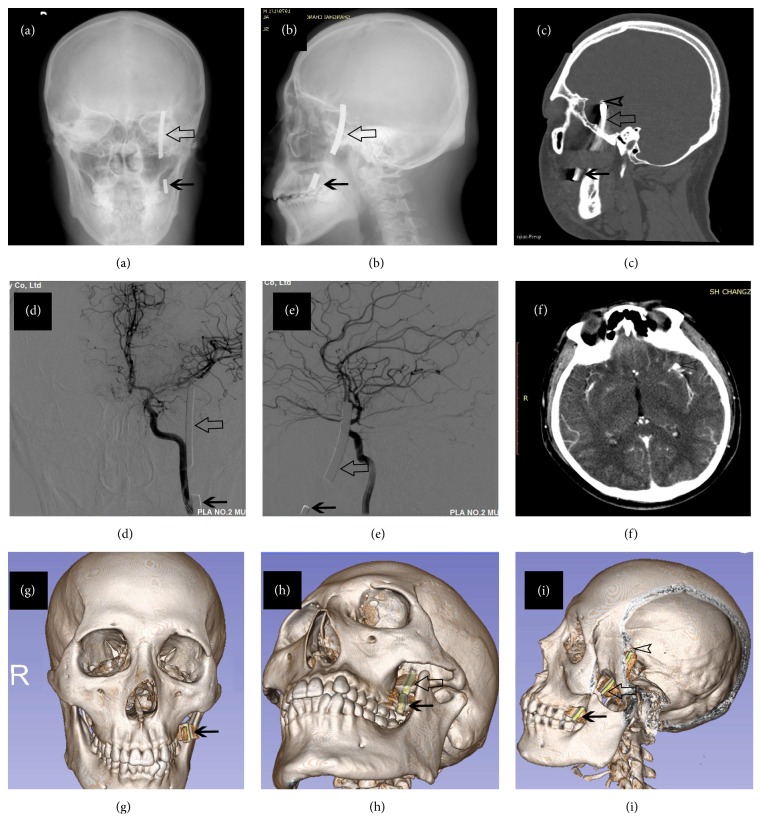
Preoperative image of foreign body (burst outer rim of a grinding wheel) in the face and middle skull base. Anterior-posterior skull radiography (a) and lateral skull radiography (b) demonstrated the short piece (simple arrow, *←*) in the face and long piece (hollow arrow, ⇦) penetrating into the middle skull base. Sagittal reconstruction of CT (c) showed the long piece penetrating into the middle cranial fossa through infratemporal fossa. A small piece of bone fragment (hollow arrowhead, *➤*) was noticed above the long piece. The bone fragment arose from the hit of the long piece on the middle skull base. DSA (d & e) proved the integrity of MCA. Axial CT revealed close relationship between the bone fragment (hollow arrow head, *➤*) and branch of MCA (f). 3D reconstruction (g, h, i) with 3D Slicer software displayed spatial correlation of two pieces of foreign body with the face and skull base. 3D, three-dimensional; CT, computed tomography; DSA, digital subtraction angiography; MCA, middle cerebral artery.

**Figure 3:**
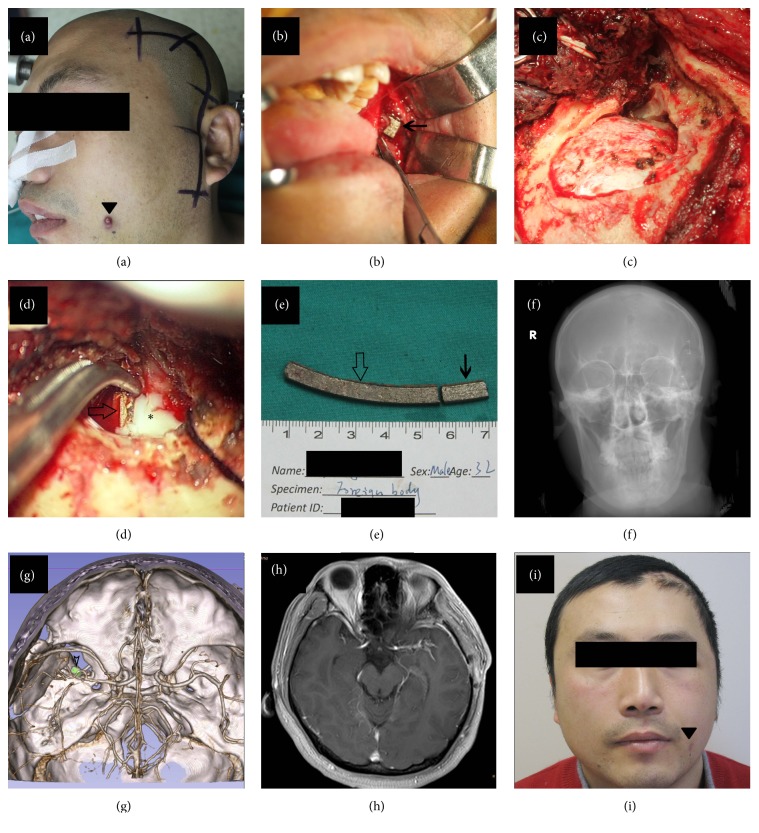
Photography showed the entry point (black triangle, ▼) of foreign body on face and incision for a frontotemporoorbitozygomatic approach (a). Short piece of foreign body (simple arrow, *←*) in the face was exposed and removed by a maxillofacial surgeon (b). The infratemporal fossa was opened to expose the long piece of foreign body (c), and it (hollow arrow, ⇦) was removed in a retrograde fashion. During the removing process, yellowish pus (asterisk, *∗*) was drained into the infratemporal fossa (d). Photography displayed short piece (simple arrow, *←*) and long piece (hollow arrow, ⇦) of the foreign body (e). Postoperative skull radiography suggested complete removal of the foreign body (f). The small bone fragment (hollow arrow head, *➤*) was left in place due to its close relationship with branch of MCA (g). Three-month follow-up MRI revealed no abscess formation (h). The patient recovered uneventfully (i). MCA, middle cerebral artery; MRI, magnetic resonance imaging.

In order to visualize the location of foreign body and its relationship with surrounding structures, three-dimensional (3D) Slicer-assisted reconstructions were conducted by a professional neuroradiologist (K. W. Han) according to presurgical imaging data. During the reconstruction, all the Digital Imaging and Communications in Medicine (DICOM) images were imported into 3D Slicer (3D Slicer 4.0~4.4; Surgical Planning Laboratory, Harvard University, USA). Segmentation of skull, foreign body, and cerebral artery was first performed with built-in modules in Slicer. Individual models of each structure were created, which could be rotated and viewed from any perspective (Figure 1(g); Figures 2(g)–2(i); Figure 3(g)).

## 3. Results

A total of 5 patients with PSBIs were identified. There were 4 males and 1 female aged 29–75 years. They were all victims of tumble or work-related accident. Four of them underwent surgical retrieval of foreign bodies and 1 patient received conservative treatment. Penetrating points as well as the surrounding neurovascular structures were clearly visualized in 3D models, assisting in the presurgical planning of optimal surgical approach and avoiding unexpected vascular injury. Details regarding patients' demographics, locations of foreign bodies, and treatment were listed in Table 1.

### 3.1. Representative Case  1

This 75-year-old female was admitted to her local hospital with complaints of headache and dizziness for 3 days. She was conscious and neurologically intact with a slightly elevated body temperature. Head CT scan revealed a low density 4 cm long foreign body extending from the left orbit to superior orbital fissure and posteriorly to the left temporal lobe (Figures 1(a)–1(c)). Brain tissue surrounding the foreign body was swollen and signs of abscess were indicated (Figures 1(d)–1(f)). The patient recalled that while she was walking in a bamboo garden, she tripped and fell forward, striking her left forehead on a bamboo stick. She did not feel any discomfort except the pain on her left upper eyelid. Symptoms of headache and fever emerged 5 days later and she was taken to the hospital by her family 8 days after the injury. Anti-infective therapies were given before she was transferred to our hospital. Careful physical examination revealed a slight skin scar on her left upper eyelid. Head DSA was performed later with no signs of vascular injuries although the bamboo stick was adjacent to the left middle cerebral artery (MCA) in the 3D reconstruction model (Figure 1(g)). Anti-infectious treatment was administrated for 12 days to control the brain abscess before surgery. During operation, an orbitozygomatic approach was adopted and the pterional craniotomy was first performed. Then superior orbital fissure was revealed after removing the great wing of sphenoid. We explored the abscess in the temporal lobe. After yellowish pus in the abscess was removed, the distal end of the bamboo stick was then visualized. We opened the dura and orbital fascia along the bamboo stick to expose its full length (Figure 1(h)). The stick was removed completely under direct visualization (Figure 1(i)) and dura was sutured in a water tight fashion. The postoperative course was uncomplicated. Broad-spectrum antibiotics were given until she was discharged free of symptoms 10 days after operation.

### 3.2. Representative Case  2

This 32-year-old man was hit by the fragments of a burst grinding wheel on his left cheek during working hours. He was taken to the local hospital complaining of headache and kept neurologically intact on physical examination. Head CT scan suggested a short piece of metal fragment locating right between the left maxillary and rami mandibulae and a long piece sticking in the left temporal lobe. Both pieces presented to be highly dense on the CT scan (Figures 2(a)–2(c)). The patient was then transferred to our department for further treatment 5 days later. DSA suggested no obvious vascular injury (Figures 2(d) and 2(e)), while axial CT scan revealed close relationship between the bone fragment and branch of MCA (Figure 2(f)). The exact location of foreign body could be visualized clearly on the 3D reconstruction model (Figures 2(g)–2(i)). Conservative management was given to control the infection and surgery was performed 15 days after the injury. During operation, short piece of the foreign body was first removed through an intraoral incision. Then a temporozygomatic approach was performed to remove the long piece, which was visualized at the infratemporal fossa after the zygomatic arch was detached and retracted downwardly with temporal muscle. Several pieces of bone fragment were found around the foreign body. After the lateral portion of infratemporal fossa was drilled off, the object was removed in a retrograde fashion. During this process, yellowish pus was drained into infratemporal fossa through the intracranial trajectory of foreign body. This trajectory was not explored and defect of dura was sealed with muscle flap (Figures 3(a)–3(e)). Postoperative skull radiography showed complete removal of the foreign body (Figure 3(f)). A small bone fragment was left in place due to its close relationship with branch of MCA, which was found to be stenotic during follow-up (Figure 3(g)). The patient was discharged 1 week after operation with sporadic focal epilepsy which was controlled well with carbamazepine, and he recovered well without abscess formation at three-month follow-up (Figures 3(h) and 3(i)).

## 4. Discussion

### 4.1. Literature Review

Clinicoradiological features of PSBIs in previous literatures were reviewed and summarized in Table 2 [2, 3, 8–24]. Most of the subjects were males (66%, 21/32) with an average age of 24 years old. 41% (13/32) of the patients were children under 10 years of age. As for the mechanism of injury, children injuries were all caused by tumbling or falling, while the injury of 4 adults resulted from suicidal or homicidal attempt, which was absent in our case series. The most common foreign body was metallic (12/32), followed by wooden (9/32) and plastic (9/32). One patient was injured by a wild deer's antler and another one was attacked by bear paws, both of which had no retained foreign bodies. As a weak area of skull, the orbit was the most common penetrating point, followed by oral or nasal cavity and maxillofacial region.

### 4.2. Diagnosis of PSBIs

After careful physical examination, proper radiological examination on the basis of patients' condition is necessary. Although the importance of head CT scan in the management of PSBIs has been emphasized in previous literatures, several instructions should be noticed. Firstly, the density of foreign bodies on CT scan varies according to their types. For example, metal presents as high density, while wood or plastics are of isodensity or low density and difficult to identify. Secondly, the density of some foreign bodies would change over time. For example, bamboo is of low density on initial CT scan, but it would be of high density later, which frequently leads to misdiagnosis (Case  1) [25]. Therefore, MRI is a useful supplement to CT in the detection of nonmetallic foreign bodies.

Angiography such as the CT angiography, MR angiography, or DSA is highly recommended for patients in suspicion of artery injuries or traumatic aneurysms [7, 26, 27]. As demonstrated in Case  2, foreign bodies may not cause immediate vascular injury sometimes but lead to cerebral vasospasm or stenosis in the long run, highlighting the importance of angiography in the setting of PSBIs.

3D modeling has been frequently used in the presurgical planning of PSBIs in previous studies [8, 11, 15–17, 19, 23, 24, 28]. 3D models can be viewed in 360 degrees, rotated, and studied from any perspective [29–31], which facilitate the diagnosis and operation to some extent [30, 31]. 3D Slicer is a free, open source software that can be used for segmentation and 3D modeling with high accuracy and reliability [30, 32]. It has been used in the management of various diseases including intracranial aneurysms, trigeminal neuralgia, and intracerebral hematomas [29, 30, 33]. PSBIs in our case series were reconstructed using 3D Slicer, in which penetrating points as well as the surrounding neurovascular structures were clearly visualized. It contributed to the presurgical planning of optimal surgical approach and avoiding unexpected vascular injury. Our findings confirmed the feasibility and reliability of 3D Slicer in the modeling of foreign objects and adjacent neurovascular structures. Moreover, the segmentation and modeling procedure using 3D Slicer allowed higher quality of visualization, better view of objects and richer information than workstation reconstruction (Figures 1(g), 2(g)–2(i), and 3(g)) [30]. To our knowledge, this is the first study to visualize PSBIs with 3D Slicer, granting preoperative surgical plan for proper approaches.

### 4.3. Treatment of PSBIs

Owing to the low incidence of PSBIs, prospective or controlled study is difficult to conduct on the limited cases. Temporary management of PBSIs constantly depends on the experience of different institutions [7, 15]. Despite the availability of some complicated guidelines for penetrating brain injury, they were mostly based on the data of scattered cases without systematic summary for PSBIs [3, 8–10]. Thus, we discussed the treatment of PSBIs based on related cases we treated and previously reported.

Operation is the major strategy for the treatment of PSBIs. Indications for surgery are retained objects, CSF leakage, fracture displacement, intracranial hemorrhage, and vascular injury [34–38]. The purpose of surgery is to remove foreign objects, decompress brain tissue, and reconstruct skull base. Generally, operation is suggested in 12 hours after PSBIs [39, 40]. However, delayed operation is not recommended until full physical and radiological examination are performed since premature surgery might lead to fatal results. Artery injury is one of the common concurrent conditions that should be identified before surgery, the presence of which will be a disaster for emergent operation [16].

In PSBIs with vascular injuries, protection of injured arteries through preoperative endovascular occlusion or intraoperative artery control would be helpful in the removal of foreign bodies [41]. In contrast, in PSBIs without vascular injuries, foreign bodies could be removed directly [7]. For some typical cases, the metal foreign bodies or bone fragments are adjacent to important structures and difficult to extract but cause no obvious symptoms. These foreign bodies could be retained in the brain (Case  2).

Surgical approach for skull base injury should be individualized according to the penetrating trajectory, location of foreign body, and accompanying vascular and brain injuries. In current study, different modifications of frontotemporoorbitozygomatic approach were adopted to manage the anterior and middle skull base injuries. By removing the superior and lateral bony orbit, we could deal with most of foreign bodies penetrating from the orbit into frontal lobe. The removal of zygomatic arch enables inferior displacement of temporalis muscle, allowing exploration of undersurface of temporal lobe. Generally, principles for skull base surgery can also be applied to PSBIs. Proximal vascular control should be first guaranteed to prevent intraoperative hemorrhage. And direct visualization of foreign object should be achieved before its removal. Usually, it is necessary to drill away the bone of skull base to expose the foreign bodies. Thorough debridement along the exposed trajectory as well as careful reconstruction of the skull base is of great significance to prevent postoperative infection and CSF leakage. However, aggressive debridement for deep seated debris should be avoided, which may be associated with increased disability and mortality [20].

Infection is the main complication of PSBIs with a reported overall rate of 64–70% and mortality rate of 14–57% [42–44]. Organic foreign body like wood or bamboo is not only the carrier, but also the best medium for infection such as brain abscess, meningitis, and cerebritis [36, 45]. In this way, organic foreign bodies should be totally retrieved, while some other deep seated foreign bodies such as small metal or bone fragments could be retained since total extraction would cause more damage. In the absence of sufficient data and definitive guidelines, the type, timing, and duration of antibiotic use remains uncertain, especially when the result of CSF culture is negative. In recent publications, prophylactic use of broad-spectrum antibiotic was suggested within 7–14 days after the injury [16, 39], while others indicated that antibiotic therapy should be administrated according to the findings of CSF culture [46]. We recommended prophylactic use of antibiotics and proper adjustment according to CSF culture results, especially for wooden objects.

## 5. Conclusions

PSBI is a rare disease with various injury mechanisms and complicated traumatic conditions. Lots of difficulties regarding the diagnosis and management of PSBIs remain to be solved. Based on our experience and review of previous studies, we suggest full physical examination and radiological evaluation before surgery. Preoperative 3D modeling with 3D Slicer could help visualize penetrating pathway and surrounding neurovascular structures in detail, granting free view from any angle and selection of optimal approach. However, caution is needed in interpreting our findings because of the limited cases. Further large scale prospective studies are required to identify the effect of preoperative 3D reconstruction on the prognosis of PSBIs, as well as the guideline for the management of PSBIs.

## Figures and Tables

**Table 1:** Demographics and clinical data for patients of PSBI.

Case	Age and gender	Mechanism of injury	Type and size of foreign body	Penetrating pathway	Symptoms and concurrent conditions	DSA findings	Interval to operation	Operation	Surgical approach	Residual fraction	Antibiotics used	Symptoms at discharge
1	75/female	Accident	Bamboo, 7 cm	Left orbit-superior orbital fissure-anterior skull base-left temporal lobe	Headache, dizziness, brain abscess	Negative	20 days	Yes	Orbitozygomatic approach and pterional craniotomy	No	Metronidazole, ceftazidime, vancomycin, linezolid, biapenem	Free of symptom
2	32/male	Accident	Grinding wheel, 6.5 cm	Left maxilla-infratemporal fossa-middle cranial fossa, cistern of lateral sulcus	Headache, brain abscess	Negative	19 days	Yes	Temporozygomatic approach	No	Linezolid	Epilepsy
3	42/male	Accident	Electrodrill, 2.2 cm	Left eyebrow-anterior cranial base-left frontal lobe	Headache, cerebral contusion	Not conducted	No operation	No	—	Yes	Linezolid, biapenem	Slight headache
4	29/male	Accident	Screw, 5.4 cm	Right orbit-anterior cranial base-right frontal lobe	Headache, blurred vision, anterior skull base fracture, oculomotor nerve injury	Not conducted	2 days	Yes	Frontotemporal approach	No	Vancomycin, linezolid	Improved vision, oculomotor nerve injury
5	40/male	Accident	Hot projective oil paint, 3.4 cm	Right orbit-anterior cranial base-right frontal lobe	Headache, blindness, right eye penetrating injury, frontal hematoma	Not conducted	4 days	Yes	Subfrontal approach	Yes	Vancomycin, ceftriaxone	Blindness

**Table 2:** Clinicoradiological features of previously reported cases of PSBI.

Author and year	Age and gender	Side/size	Material	Mechanism of injury	Penetrating pathway	Symptom	Surgical approach	Infection	Outcome
Matsumoto et al., 1998	3/female	Left/3 cm	Plastic chopstick	Falling	Optic canal	Blindness	Craniotomy	NK	Blindness
Matsumoto et al., 1998	57/male	Right/2 cm	Wooden chopstick	Stick into chopstick	Optic canal	Blindness	Craniotomy	NK	Blindness
Hebecker, 2009	32/male	Left/no foreign body	Wild deer's antler	Accident	Left orbit floor/lateral orbit	Coma	Bifrontal intradural neurosurgical approach	No	Free of symptom
Ishikawa et al., 2000	4/male	Left/6 cm	Wooden chopstick	Falling	Preauricular area to posterior fossa	Fever, headache	Suboccipital craniectomy	Brain abscess	Ataxia and dysmetria
Matsuyama et al., 2001	1/male	Right/2.5 cm	Wooden chopsticks	Falling	Superior orbital fissure	Swollen eye	Transcranial approach	Meningitis	Free of symptom
Maruya et al., 2002	56/female	Left/1 cm	Bamboo	Tumbling	Temporal (lateral orbit)	Drowsiness	Left frontotemporal craniotomy and orbitozygomatic osteotomy	No	Lateral gaze in the left eye
Hayashi et al., 2003	71/female	Right/no foreign body	Bear claws	Animal assault	Frontal sinus	Lacerated face, CSF fistulas	Frontal craniectomies to repair CSF fistulas	No	Free of symptom
Nishio et al., 2004	6/female	Right/2.5 cm	Wooden chopstick	Falling	Frontal (orbital roof)	Fever, headache, neck stiffness	Craniotomy	Brain abscess	Free of symptom
Kim et al., 2005	1/male	Right/2 cm	Metallic chopstick	Falling	Frontal (orbital roof)	Crying	Craniotomy	No	Free of symptom
Tsao et al., 2006	45/male	Left/2.5 cm *∗* 2.5 cm	Plastic chair glide	Be assaulted	Sinonasal cavity, anterior skull base	Ptosis, impaired vision	Transnasal endoscopic exploration and removal of the foreign body	No	Anosmia and diplopia
De Tommasi et al., 2006	20/male	Right/10 cm	Screwdriver	Falling	Right maxilla	Scotoma in the left eye, CSF leakage	Left pterional approach	NK	Restored vision
Park, 2006	9/female	Right/NK	Wooden chopstick	Falling	Frontal (orbital roof)	Swollen eye	Craniotomy	No	Free of symptom
Park, 2006	1/male	Left/NK	Metallic chopstick	Falling	Frontal (orbital roof)	Swelling, left hemiparesis	NK	No	Left mild hemiparesis
Park, 2006	5/male	Left/NK	Metallic chopstick	Falling	Frontal (orbital roof)	Swollen eye	NK	No	Free of symptom
Park, 2006	2/male	Left/NK	Metallic chopstick	Falling	Frontal (orbital roof)	Swollen eye	NK	No	Free of symptom
Nitsch et al., 2007	22/male	Right/2 cm	Nail-gun	Accident	Right upper jaw to temporal lobe	Intracerebral haematoma	Simple extraction of the nail without a craniotomy	NK	NK
Hiraishi, 2007	5/female	Left/3.5 cm	Plastic chopstick	Falling	Frontal (orbital medial)	Fever, headache, neck stiffness	Craniotomy	Meningitis, brain abscess	Hyposmia
Kawada, 2009	5/female	Left/3 cm	Wooden chopstick	Falling	Frontal (orbital roof)	Swollen eye	Craniotomy	Brain abscess	Free of symptom
Mitilian, 2009	4/male	Right/11 cm	Wooden chopstick	Falling	Superior orbital fissure	Mild confusion, vomiting	Craniotomy	Brain abscess	Mild dysmetria of the arm
Wieland et al., 2010	58/male	Left-sided/NK	Wooden stick	Accident	Maxillofacial to ethmoid roof	Fever, headache, nausea, vomiting, mental status changes	Endoscopic repair of the skull base defect	Intracranial infection	NK
Hettige et al., 2010	38/female	Left/NK	Plastic chopstick	Stumble	Posterior wall of the oropharynx to jugular foramen into the posterior fossa	Nystagmus, left 9th, 10th, and 12th cranial nerves palsy, quadrantanopia	Left occipital craniotomy, retrosigmoid craniectomy	No	Left 9th, 10th, and 12th cranial nerves palsy
Sweeney et al., 2011	31/male	Right/NK	Knife	Suicidal attempt	Lower jaw to anterior skull base	Pain	Combined right pterional and interhemispheric craniotomies	No	NK
Sweeney et al., 2011	21/female	Right/NK	Knife	Homicidal attempt	Left orbit to middle skull base	Emesis, seizure	Left cranioorbitozygomatic approach	No	NK
Yonezawa, 2011	28/male	Left/NK	Plastic chopstick	Falling	Frontal (orbital medial)	NK	Craniotomy	No	Free of symptom
Arslan et al., 2012	13/male	Right/18 cm	Iron bar	Falling	Right orbit and superior orbital fissure	Coma (GCS3)	Right frontoparietal craniotomy	No	Died
Shin et al., 2012	38/male	Left/14 cm	Plastic chopstick	Falling	Superior orbital fissure	Blindness, swelling, numbness around eye	Removal of chopstick without craniotomy	No	Blindness
Katayama et al., 2013	18/male	Right/12 cm	Metal rod	Falling	Frontal (subzygomatic bone)	Vomiting	NK	NK	No neurological deficit
Wang et al., 2013	35/male	Left/18 cm	Steel	Falling	Left maxillary sinus	Limited movement of neck	Craniotomy	No	Left facial palsy
Skoch et al., 2013	35/female	Medial/NK	Electric toothbrush	Accident	Temporal (lateral orbit)	Blurred vision, pain	Modified frontotemporal orbitozygomatic craniotomy	No	Improved pain and vision
Deveer et al., 2013	43/male	Right/5.6 cm	Plastic pen	Falling	Right orbit	No	NK	No	Free of symptom
Yamasaki et al., 2013	4/female	Left/6.5 cm	plastic chopstick	Falling	Frontal (orbital roof)	Swelling	NK	Meningitis, encephalitis	Free of symptom
Williams et al., 2014	56/male	Left/NK	Speargun	Suicidal attempt	Submandibular region, oral cavity	Coma	Large craniotomy	Cerebritis	Died

ACA, Anterior Cerebral Artery; CSF, Cerebrospinal Fluid; GCS, Glasgow Coma Scale; ICA, internal carotid artery; NK, not known.
